# Artificial intelligence for animal science: from applications to integrated knowledge systems

**DOI:** 10.1093/af/vfaf054

**Published:** 2025-12-18

**Authors:** Mutian Niu, Chuanyi Guo, Victor E Cabrera

**Affiliations:** Animal Nutrition, Institute of Agricultural Sciences, Department of Environmental Systems Science, ETH Zürich, 8092 Zürich, Switzerland; Animal Nutrition, Institute of Agricultural Sciences, Department of Environmental Systems Science, ETH Zürich, 8092 Zürich, Switzerland; Department of Infectious Diseases and Public Health, Jockey Club College of Veterinary Medicine and Life Sciences, City University of Hong Kong, Hong Kong SAR, China; Department of Animal and Dairy Sciences, University of Wisconsin-Madison, Madison, WI 53706

**Keywords:** artificial intelligence, integrated systems, knowledge co-producer, sustainable agriculture

Implications

- AI is shifting from discrete tools to a system-level integrator, requiring a holistic approach to manage farm ecosystems rather than isolated disciplines.
- Advanced AI transforms farms into real-time living laboratories, accelerating knowledge creation and positioning AI as a co-producer of scientific discovery.
- The next frontier is a multiscale vision for AI, integrating data across molecular, animal, herd, and environmental levels to sustainably manage agricultural ecosystems.
- Responsible AI deployment requires co-developed frameworks for ethics, data governance, and equity to protect animal welfare, ensure farmer agency, and build trust.
- Practitioners should view integrated AI as a long-term investment. Emerging evidence shows measurable productivity and environmental gains over 3–5 year periods, but return of investment timelines will vary by farm.

## Introduction

Animal science is at a critical juncture, faced with the challenge of providing sustainable nutrition for a growing global population. Against this backdrop, Artificial Intelligence (AI) has emerged as a powerful transformative force, offering the potential to enhance efficiency, improve animal welfare, and reduce environmental footprints (Distante et al., 2025). To date, the application of AI has largely taken the form of discrete, stand-alone solutions, with specific tools successfully addressing challenges in areas such as health monitoring and feed optimization. This toolbox paradigm has yielded significant gains and demonstrated the clear value of data-driven management (Cabrera, 2024; Menezes et al., 2024).

However, a fundamental leap in the technology itself now invites a more ambitious vision. Whereas early AI was primarily analytical, tasked with identifying patterns in existing data, contemporary generative approaches, driven by large language models, vision models, and AI agents (systems that can perceive their environment and take autonomous actions), have begun to demonstrate synthetic reasoning and proactive planning capabilities (Li et al., 2025). Such a qualitative progression, from analytical to generative AI, has the potential to transform the concept of an integrated knowledge system from a distant aspiration into an attainable reality.

It is this transition and its profound implications that this review seeks to explore. We articulate how AI is evolving beyond a passive farm management tool to become an active co-producer of scientific knowledge and a cross-disciplinary system integrator. This article argues the revolutionary impact of AI in animal science lies not in isolated tools but in its emerging role as a systemic driver, with the potential to advancing the entire field from disparate applications toward an integrated knowledge and decision-making ecosystem. However, realizing this vision requires addressing fundamental challenges in validation, reproducibility, and responsible deployment.

## AI in Animal Science: From Data Points to Digital Ecosystems

**Figure 1.**
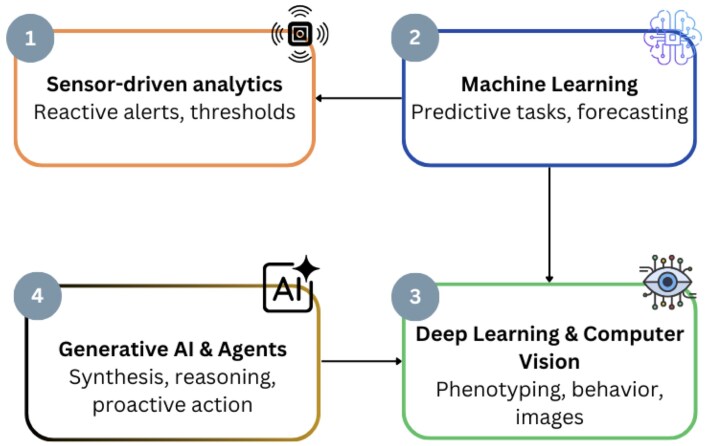
Evolution of AI in animal science from data points to digital ecosystems.

To understand the paradigm shift currently underway, it is necessary to review the evolution of AI in animal science from both a technological and a conceptual standpoint (Ghavi Hossein-Zadeh, 2025). The application of AI has progressed through four identifiable stages, each representing a significant increase in analytical sophistication and operational autonomy (Figure 1). The initial phase was characterized by sensor-driven analytics, where technology was used to monitor discrete variables, such as an animal’s temperature or activity level. These systems were largely reactive, designed to trigger passive alerts when a predefined threshold was crossed, signaling a potential issue for human intervention.

A significant advancement came with the application of machine learning (ML). As sensor data accumulated, ML algorithms enabled a shift from simple reactive alerts to predictive tasks (García et al., 2020). These models could analyze historical data to forecast outcomes, such as the likelihood of disease, predict milk production, or optimize feed formulations to improve management efficiency.

The next leap in capability was driven by the advent of deep learning, particularly through computer vision. This technology unlocked the potential to analyze complex, unstructured data, such as images and video, enabling more sophisticated and noninvasive phenotyping (Okinda et al., 2020). Through image analysis, it became possible to monitor intricate animal behaviors, estimate body weight, and even identify individual animals within a herd automatically. The current frontier marks another fundamental shift, defined by the rise of generative AI and agent-based systems. This fourth stage moves beyond analysis and prediction into the realm of synthesis and autonomous action. Powered by large language and vision models, these systems possess advanced capabilities for reasoning, simulation, and proactive decision-making (Ferreira and Dórea, 2025).

More importantly, this technological progression has enabled an equally profound conceptual evolution. Early AI applications were constrained by both a scarcity of on-farm data and the computational power needed to process it. The proliferation of Internet of Things sensors solved the data generation problem but often resulted in information being stored in isolated data silos. Concurrently, the rise of cloud computing and deep learning provided the immense power required to analyze these vast and disparate datasets. The convergence of these two trends, which are widespread data generation and massive computational capacity, has made a holistic systems approach practically achievable for the first time (Kaur et al., 2023). This represents a fundamental change in mindset, moving from reactive, problem-specific solutions (e.g., identifying a sick animal) to proactive, systems-level optimization (e.g., adjusting the herd’s environment to minimize future disease risk). This shift from viewing the farm as a collection of data points to understanding it as an interconnected digital ecosystem provides the foundation for AI’s emerging role as a true system integrator.

## The Core Paradigm Shift: AI as a System Integrator

**Figure 2.**
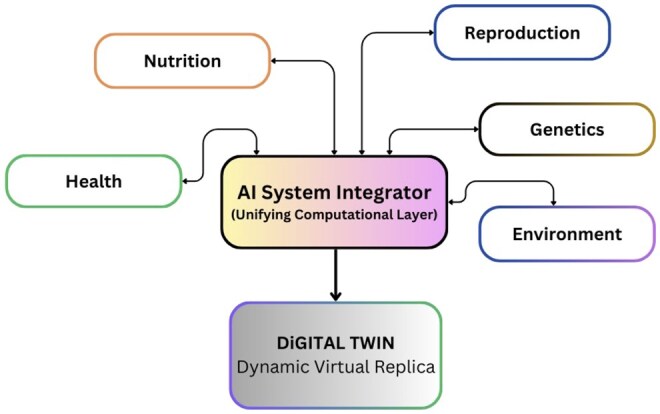
AI as a system integrator connects siloed domains of dairy science into a digital twin framework enabling proactive, cross-domain optimization.

The next revolutionary step for AI in animal science is its function as a system integrator (Figure 2) capable of understanding of complex interactions. For example, a change in a feed ration may have cascading effects on an animal’s reproductive health and methane emissions, but these connections can be difficult to quantify when data is not integrated. AI provides a unifying computational layer. This layer should not be envisioned as a single, monolithic model (e.g., a specific GPT), but rather as a sophisticated architecture that allows multiple, specialized AI models (such as computer vision models, forecasting models, and generative agents) to interoperate. This is that architecture ingests diverse data streams from these areas, placing them within a single analytical framework to reveal previously hidden interconnections and enable proactive, cross-domain optimizations.

The ultimate expression of this integrated approach is the digital twin, a dynamic, virtual replica of a physical entity (such as a single animal or an entire farm) defined by a continuous, bidirectional flow of data between the physical and virtual worlds (Escribà-Gelonch et al., 2024). Real-time information from sensors on the physical entity constantly updates the virtual model, while simulations and optimizations run on the model provide decision support to directly guide actions in the real world. This capability allows managers to conduct complex hypothetical analyses and elevates farm management from descriptive, backward-looking assessments to proactive, predictive optimization.

To make this abstract concept concrete, the pioneering effort to connect the DairyBrain project (Cabrera et al., 2020; Ferris et al., 2020; Cabrera, 2024) at the University of Wisconsin–Madison with the Ruminant Farm Systems (RuFaS) model (Kebreab et al., 2019; Hansen et al., 2021; Li et al., 2023) illustrates the practical pathway toward this vision. DairyBrain functions as a data integration hub, using an application programming interface to automatically pull, clean, and standardize data from previously disconnected on-farm software systems, including herd management, feeding, and milking parlor data (Wangen et al., 2021). The RuFaS model, in turn, is a next-generation, open-source simulation tool designed to assess how different management practices affect systemic outcomes like economic profitability, environmental footprint, and animal welfare. The explicit goal of the collaboration between these projects is to create a data pipeline where the unified stream from DairyBrain can feed the RuFaS model, thereby laying the groundwork for a complete loop from raw data collection to sophisticated, cross-disciplinary knowledge generation.

This level of deep integration and virtualization gives rise to a powerful new concept: the computational phenotype. This is not merely a collection of metrics but a high-dimensional, data-driven expression of the animal, derived from the unification of its extensive behavioral, physiological, and production data. In practice, this expression manifests as a series of quantifiable novel derived traits (Brito et al., 2025). This computational phenotype can capture subtle, complex patterns, such as minor behavioral changes preceding clinical disease, resilience calculated from the raw data of milk yield fluctuations or fertility traits defined through activity monitoring data, that are difficult or impossible to measure with traditional methods. It provides a new language for animal breeding and management, suggesting a future where selection goals may include not only traditional genetic traits but also the optimization of these comprehensive, data-defined derived traits.

## From Proof-of-Concept to On-Farm Reality

While the vision of AI as a system integrator is compelling, translating it from research prototypes to widespread on-farm reality requires navigating a significant gap. The path to adoption is not merely a matter of refining algorithms or scaling infrastructure. It involves overcoming fundamental barriers that determine whether these powerful tools will be accepted, trusted, and used effectively in the real world (Dibbern et al., 2024). Addressing these challenges within agriculture, informed by the valuable lessons learned from other high-stakes industries, provides a constructive path forward.

### The triple barrier to adoption

The widespread adoption of integrated AI systems is hindered by an interconnected set of technical, socioeconomic, and human-centric barriers (Baldin et al., 2025). The technical challenges are rooted in the lack of data standards and interoperability between systems from different vendors, which prevents the seamless data fusion necessary for a true digital twin (Cabrera et al., 2025). Furthermore, while low-earth-sorbit satellite providers are solving historic issues of low speed and latency, they introduce new adoption barriers, including high initial hardware costs, reliance on proprietary technology, and a lack of local technical support (McMahon et al., 2025).

From a socioeconomic perspective, the high initial investment and switching costs are significant deterrents for farmers, particularly as the return on investment has historically been difficult to quantify. However, concrete, longitudinal evidence is beginning to emerge. For example, a life-cycle assessment of intensive dairy goat farms implementing an integrated smart-farming platform found significant environmental benefits over 4 years, including an 11% reduction in greenhouse gas emissions and a 9–16% reduction in other impacts. These gains were driven by improved resource efficiency, lower replacement rates (−20%), and higher annual milk productivity (+11%; Pardo et al., 2022). Despite this emerging evidence, adopting these systems also requires new technical skills, creating a potential labor gap that the industry must address.

The third, and perhaps most critical, barrier relates to human trust and governance (Barton et al., 2025). Farmers are often cautious about ceding critical decision-making authority to opaque black box algorithms. This issue is particularly acute with the deep learning models that power many advanced AI tools. Because of their complex internal architecture, these models often suffer from poor interpretability, making it difficult or impossible for a user to understand why a particular recommendation was made. While emerging generative models offer “reasoning” capabilities, their capacity to accurately explain the internal, latent-space decisions of complex deep learning models is, in itself, a highly technical and unresolved research challenge. This lack of transparency erodes trust and is compounded by significant concerns regarding data ownership, privacy, and security in the absence of clear governance frameworks.

### Lessons from other high-stakes industries

These challenges, while significant, are not unique to agriculture. By examining the deployment of complex AI in other high-stakes sectors, particularly precision medicine and autonomous vehicles, valuable lessons can be learned. In precision medicine, the implementation of AI has been hampered by algorithmic bias stemming from unrepresentative patient data, which can perpetuate health disparities. The black box problem has also slowed adoption by clinicians who, much like farmers, require explainable and interpretable outputs to build the trust necessary for high-stakes decisions (Ricci Lara et al., 2022).

The rollout of autonomous vehicles offers particularly relevant insights. First, it has demonstrated that existing legal frameworks are often inadequate for new technologies (Olayode et al., 2023). Traffic laws and insurance models were built on the assumption of a human driver as the responsible agent. AI shifts this responsibility to a technological system, necessitating the creation of entirely new regulations for liability, rather than simply attempting to retrofit old rules designed for human actors. Second, early visions of autonomous transport often involved creating new, smart infrastructure, such as roads embedded with sensors for vehicle-to-infrastructure communication. However, the immense cost and complexity of this approach have proven impractical and the more successful path has been to develop AI that can navigate the existing, imperfect infrastructure. This provides a powerful lesson for animal agriculture: the goal should not be to require farmers to replace their entire infrastructure, but to develop AI systems that can integrate with and enhance the equipment and facilities already in place.

These cross-industry experiences provide a clear roadmap for animal science. The challenges in precision medicine underscore the absolute necessity of developing AI models using diverse datasets that represent a wide range of breeds and production systems to avoid biased outcomes. The lessons from autonomous vehicles highlight the importance of establishing new legal frameworks for data ownership and liability, as well as focusing on integration with existing farm systems. Most importantly, these fields converge on a single, critical point: the necessity of a co-design process. To ensure that AI tools are practical, valuable, and trusted, farmers, veterinarians, ethicists, and social scientists must be involved from the earliest stages of development, not merely consulted after the fact. This collaborative approach is the most effective way to bridge the gap between a promising concept and a transformative on-farm reality.

## The Next Frontier: AI as a Co-producer of Scientific Knowledge

**Figure 3.**
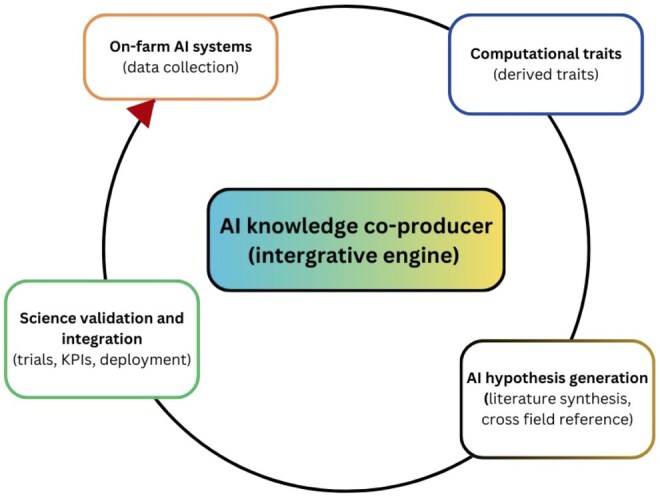
AI-science feedback loop: from practice to discovery and back.

Beyond its role as a sophisticated tool for farm management, AI is poised to enter a potentially transformative new phase: as an active partner in the scientific discovery process (Zhang et al., 2025). This hypothesized evolution marks a fundamental shift from using AI to analyze data within existing research paradigms to leveraging it to assist in generating novel hypotheses, uncover previously invisible biological phenomena, and potentially influence the scientific method itself. In this new capacity, AI is not merely processing information but is beginning to function as a co-producer of knowledge, augmenting human intellect and potentially accelerating the pace of innovation (Figure 3).

The derived traits, generated by the so-called computational phenotype, serve as the foundation for this new frontier. Rather than simply automating the measurement of known traits, AI’s role here is to analyze these complex datasets to identify subtle, multivariable patterns that are beyond human perception or traditional statistical methods. This capability has the potential to transform the practice of phenotyping, moving it from a hypothesis-led approach focused on predefined traits to a data-driven, exploratory process. Through this process, novel and emergent properties of the biological system can be discovered, including the latent functions and competencies of biological networks. These are adaptive behaviors that only manifest under specific conditions, and their discovery deepens our understanding of the complexity of living systems.

This ability to uncover novel patterns is complemented by an even more profound capability: the autonomous generation of new and testable scientific hypotheses. Early demonstrations suggest several pathways for this capability. On one hand, AI systems can act as a tireless research collaborator by automating the synthesis of vast amounts of scientific literature to identify knowledge gaps and propose new lines of inquiry. For example, prototype systems such as Google’s AI co-scientist, deploys multiple specialized agents working together to generate, critique, and refine research proposals. While some preliminary reports suggest these systems can outperforming unassisted human experts in some cases, such claims are based on preprint studies and require independent, peer-reviewed validation (Gottweis et al., 2025). On the other hand, AI is also emerging as an idea generation tool that may help explore the latent space of scientific knowledge, the realm of logically plausible but as-yet-unarticulated ideas. Frameworks like FieldSHIFT use large language models to bridge concepts between scientific fields with deep structural symmetries, generating unexpected but scientifically valid new hypotheses (O’Brien et al., 2024). Together, these potential capabilities hint at a future where AI moves from a passive analytical tool toward an active engine for scientific innovation.

These advancements culminate in a powerful, self-reinforcing feedback loop between on-farm practice and scientific research. The integrated AI systems deployed on farms are generating the massive, high-quality datasets that constitute the computational phenotypes of animals. This real-world data becomes the raw material for research AI to analyze and discover new, more refined phenotypes. These newly discovered traits can then be integrated back into the on-farm AI systems as the next generation of key performance indicators for monitoring and management. This cycle transforms the farm from a passive site of research application into an active, real-time laboratory, blurring the lines between basic science and industry practice and dramatically accelerating the cycle of knowledge creation and technological iteration.

While these opportunities are promising, the generative capabilities discussed are nascent and carry significant risks, such as algorithmic hallucination, the propagation of biases from training data, and a lack of model reproducibility (Branda et al., 2025). These challenges necessitate a shift in scientific practice: all AI-generated hypotheses must be treated as preliminary and require rigorous human validation and critical oversight.

Ensuring these insights meet rigorous scientific standards is paramount. This requires the development and adoption of new validation frameworks. Best practices should include the pre-registration of AI-driven hypotheses before testing, rigorous validation through controlled experimental workflows, cross-validation against independent datasets, and the development of community benchmarks or challenge datasets to transparently assess model performance (Caminero et al., 2025). Adopting such robust validation practices is essential to build trust and separate plausible-sounding “hallucinations” from genuine, reproducible scientific discovery.

## The Socio-ethical Dimensions of AI-Driven Animal Science

As AI becomes more deeply integrated into animal science, its influence extends far beyond technical and economic metrics, raising profound social and ethical questions that will shape the future of agriculture. The design and deployment of these technologies are not neutral acts, as they reflect underlying values and will carry important implications for animal welfare, farmer agency, and the equitable distribution of power within the global food system. A deliberate and proactive engagement with these dimensions is essential to ensure that this technological transition supports outcomes aligned with sustainability, animal welfare, and social equity.

### The duality of AI: welfare, governance, and equity

A central ethical issue is the dual potential of AI in animal welfare. On one hand, AI offers the promise of unprecedented individualized care. Through continuous, noninvasive monitoring, AI-powered systems can detect early signs of disease, distress, or discomfort. This enables timely interventions that can significantly reduce animal suffering and improve overall welfare. This technology can provide a level of vigilance that is difficult to achieve with human labor alone, ensuring that animals receive prompt attention when needed. On the other hand, there is a significant risk that AI could be used to hyper-optimize the efficiency of intensive, low-welfare production systems, thereby entrenching and even expanding their scale (Weary and von Keyserlingk, 2023). This could accelerate a trend toward systems that are increasingly opaque, where animals are reduced to data points managed by algorithms, and the role of human care and direct animal interaction is minimized. Without safeguards, this trajectory could incentivize expansion of high-efficiency, low-welfare systems, where the economic advantages conferred by AI are used to justify systems that inherently compromise animal well-being.

Beyond the animals themselves, the deployment of AI raises critical questions of governance, equity, and farmer agency. Data governance is a core concern, raising questions about who owns, controls, and benefits from the vast quantities of data generated on farms (Cue et al., 2021). This challenge manifests differently across regions: the EU’s 2024 Data Act, for instance, has regulatory gaps in its legal definition of a “user” that may exclude farmers from their data in common AI service models. In contrast, the United States lack of binding federal regulation creates different gaps, where power imbalances allow for data lock-in strategies to be enforced through complex contracts (Uyar et al., 2024). If control over agricultural data and the AI systems that analyze it becomes concentrated in the hands of a few large technology corporations, it could create new forms of dependency, shifting power away from farmers and toward these external entities. This also raises the risk of widening the existing digital divide. Without intentional efforts to ensure equitable access, the benefits of AI may flow primarily to large, well-capitalized corporate farms, while smallholder farmers, who are critical to global food security, could be left behind, threatening the diversity and resilience of the agri-food system.

### Ethical principle: openness, empowerment, and co-design

However, these negative outcomes are not inevitable. The technological path forward is a matter of choice, and an alternative centered on fairness, openness, and empowerment is both possible and necessary. In contrast to closed, proprietary platforms, open-source initiatives provide powerful, transparent toolkits that allow researchers and farmers to innovate and adapt solutions to their specific needs without vendor lock-in. The development of the Bovine Heat Detection and Analysis Tool (BovHEAT) illustrates this approach. It is a validated, open-source software that processes data from activity monitoring systems in dairy cattle, helping to accelerate research and improve reproducibility in reproductive management (Plenio et al., 2021). New governance models, such as community-owned data trusts or farmer cooperatives acting as data stewards, offer a mechanism for producers to manage their collective information as a shared asset, ensuring they retain control and derive value from their own data. Practical examples of such frameworks include the U.S. “Ag Data Transparent” certification, which provides a voluntary whitelist for trustworthy data partners, and the development of “model contractual terms” in the EU (Ryan et al., 2024).

Furthermore, AI tools can be specifically designed to empower smallholders, and a review of digital livestock tools in India and Kenya provides several promising examples. E-extension platforms like iCow in Kenya deliver crucial animal husbandry advice on feeding and disease control directly to farmers via accessible SMS-based services. A different model of indirect empowerment is seen with tools like Herdman in India, which is used by dairy cooperatives to monitor and improve the quality of veterinary and artificial insemination services provided to smallholders. For more commercially-oriented farmers, herd management platforms like Farmtree in India offer sophisticated, animal-specific analytics to optimize economic performance. These cases illustrate a range of strategies, from direct knowledge transfer to indirect service improvement, for deploying AI to support small-scale producers (Daum et al., 2022).

These examples demonstrate that technology can be a force for equity rather than a driver of inequality. They make it clear that ethical considerations, such as animal welfare, farmer agency, data sovereignty, and equity, cannot be treated as afterthoughts. These principles must be integrated as core design elements from the very beginning of the development and deployment process, transforming ethics from a passive critique into an active, constructive directive for innovation. In this way, socio-ethical principles directly shape whether AI fulfills its role as both a system integrator and a co-producer of scientific knowledge in animal science.

## The Road Ahead: A Multiscale and Sustainable Vision

Building upon the foundations of system integration and AI-driven scientific discovery, the future development of AI in animal science should be guided by a comprehensive vision that is multiscale, multispecies, and fundamentally oriented toward the goal of sustainability (Rosati, 2024). This trajectory is not simply a technological extension of current capabilities but a necessary response to the global challenges of food security and climate change. It positions AI as a potentially indispensable partner in shaping a more resilient and responsible future for agriculture. Realizing this vision will require advances in data integration, computational efficiency, with a focus on strategies such as model distillation, edge computing, and federated learning to mitigate the environmental footprint of large models and overcome current technical and institutional limitations.

A significant frontier for research will involve moving beyond the current paradigm of single-species models. The development of cross-species foundational models, trained on data from a wide variety of animals, could enable the discovery of conserved biological principles that govern health, physiology, and behavior across different species. However, unequal data availability and quality across species (e.g., dairy cattle vs. small ruminants or aquaculture) may bias these models, underscoring the need for targeted investment in diverse datasets.

**Figure 4.**
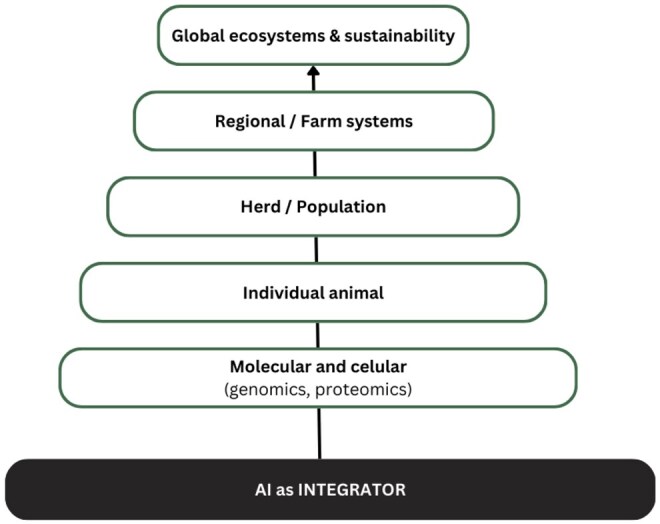
Multiscale vision for AI in animal science.

At the same time, the scale of integration must expand to achieve a truly multiscale understanding of animal production. This means creating systems that can seamlessly connect data from the molecular and cellular levels, such as genomics and proteomics, to the individual animal, the herd, the farm, and ultimately to the regional and global ecosystems in which they are situated. Such a holistic view is essential for managing the complex interplay between animal agriculture and broader environmental health (Figure 4). For example, linking genomic markers of feed efficiency to herd-level methane mitigation strategies illustrates how molecular data can inform sustainability at farm and ecosystem scales.

Ultimately, AI must be positioned not merely as a tool for enhancing economic productivity, but as a cornerstone of sustainable agriculture. By enabling unprecedented levels of precision, AI can optimize the use of critical resources like feed, water, and energy, thereby minimizing waste and reducing the environmental footprint of livestock farming, including greenhouse gas emissions, nitrogen cycling, and water consumption. In an era of increasing climate volatility, AI can also help build more resilient food systems by providing farmers with adaptive management strategies based on accurate predictions of extreme weather events. The deep integration of AI technology with clear sustainability objectives is therefore critical to ensuring that these powerful tools serve the long-term, collective well-being of animals, people, and the planet. Moreover, sustainability must be understood as encompassing not only environmental outcomes but also social and ethical dimensions such as equity, farmer livelihoods, and consumer trust, linking back to the principles outlined in Section 6.

## Conclusion

AI in animal science is moving from fragmented tools toward integrative systems that connect disciplines, generate new knowledge, and advance sustainability. Its transformative potential lies in four dimensions: serving as a system integrator of nutrition, reproduction, health, genetics, and environment; acting as a co-producer of scientific discovery through computational phenotypes and hypothesis generation; embedding socio-ethical principles to protect welfare, agency, and equity; and driving a multiscale vision that links molecular biology to global food systems. Realizing this potential, however, requires overcoming bottlenecks in data governance, interoperability, dataset diversity, energy demands, and reproducibility. Progress depends on distinct responsibilities: researchers must ensure rigor and innovation, extension specialists must build trust and local adaptation, policymakers must set fair governance frameworks, and industry must commit to open, farmer-centered models. The future of AI in animal science will not be determined by algorithms alone, but by the values we embed in them—whether they become mere instruments of efficiency or catalysts for resilient, humane, and sustainable livestock systems.

## Author Biographies

**Dr. Mutian Niu** is an Assistant Professor of Animal Nutrition at ETH Zurich’s Institute of Agricultural Sciences, Department of Environmental Systems Science. His research advances sustainable livestock production by integrating hypothesis-driven experiments with data science, focusing on ruminant nutrition, nutritional physiology, greenhouse gas mitigation, nitrogen metabolism, heat stress alleviation, statistical modeling, precision livestock farming, and exhalomics/breathomics for noninvasive monitoring of animal health and emissions. Prior to ETH Zurich, Dr Niu served as an Assistant Professor at the University of Pennsylvania’s School of Veterinary Medicine and as a Data Scientist at Farmer’s Business Network. He earned his Ph.D. in Animal Biology and M.S. in Statistics from the University of California, Davis; an M.S. in Animal Science from Pennsylvania State University; and a B.S. in Animal Science from China Agricultural University. Dr Niu has secured > USD 3.4 million in grants, including projects on net-zero emissions pathways. He has authored >50 publications in journals like the Annual Review of Animal Biosciences, Journal of Dairy Science, Global Change Biology, Animal, etc He serves as Co-Chair of the EAAP Precision Livestock Farming and AI for Animal Science Working Group, Guest Editor for Animal Frontiers, and a member of FAO LEAP and the Global Research Alliance on Agricultural Greenhouse Gases. His interdisciplinary approach bridges academia and industry to address global challenges in food security and climate change.

**Chuanyi Guo** is a Ph.D. candidate in the Department of Infectious Diseases and Public Health at the Jockey Club College of Veterinary Medicine and Life Sciences, City University of Hong Kong. He received his bachelor’s and master’s degrees in computer science and technology from Harbin Institute of Technology (HIT) and the Chinese University of Hong Kong (CUHK) in 2022 and 2023, respectively. His current research focuses on the application of computer vision and sensor-based machine learning for animal behavior and welfare, as well as the development of cloud-edge collaborative systems.

**Dr. Victor E. Cabrera** is a leading expert in data-driven decision support for dairy farm management, integrating applied research, interdisciplinary approaches, and participatory methods to develop practical, user-friendly tools that enhance farm profitability, environmental stewardship, and long-term sustainability. He leads the Dairy Brain project, a pioneering initiative in data integration and analytics for dairy farming, and actively contributes to the RuFaS project, which advances resilient and climate-smart agricultural systems. Dr Cabrera and his research team have developed over 50 decision support tools, published more than 110 refereed articles and 12 book chapters, and his work has had a global impact through research and extension. He has presented at over 140 scientific sessions and delivered more than 400 extension talks across Wisconsin, the United States, and internationally. Over the past 15 years, Dr Cabrera has secured over $5.5 million in funding to support his research and extension initiatives. His contributions have been recognized with numerous prestigious awards, including the Vilas Associate Award, Excellence in International Activities, the DeLaval Dairy Extension Award from the American Dairy Science Association, the Vilas Faculty Mid-Career Investigator Award, and several other distinctions from UW-Madison, the Wisconsin Association of County Agricultural Agents, the University of Florida, and the American Dairy Science Association.
